# Repercussions of Symbiotic Bacteria Associated with Entomopathogenic Nematodes and Their Biogenic Silver Nanoparticles on Immune Responses at Root-Knot Nematode Suppression

**DOI:** 10.3390/microorganisms14010092

**Published:** 2025-12-31

**Authors:** Rehab Y. Ghareeb, Shawky M. Eid, Hanan Alfy, Mohamed H. Elsheikh

**Affiliations:** 1 Plant Protection and Biomolecular Diagnosis Department, Arid Lands Cultivation Research Institute, City of Scientific Research and Technological Applications (SRTA-City), Alexandria 21934, Egypt; 2 Top Chemicals Company for Pesticides, Alexandria 21934, Egypt; 3 Field Crop Pests Department, Plant Protection Research Institute, Agriculture Research Center, Giza 12619, Egypt; 4 Plant Production Department, Arid Lands Cultivation Research Institute, City of Scientific Research and Technological Applications (SRTA-City), Alexandria 21934, Egypt

**Keywords:** root-knot nematodes, acetylcholine esterase genes, Q-PCR, eco-friendly nematicide, biogenic nanoparticles

## Abstract

Root-knot nematodes (RKNs) of the *Meloidogyne* genus impact various plants, including crops, fruits, and vegetables. Few chemical control options exist globally, and many nematicides are banned due to health and environmental risks. This study tested a new nematicidal agent, the symbiotic bacterium *Xenorhabdus indica*, which was molecularly identified (PV845100). Cell-free culture supernatants of *Xenorhabdus* spp. and their biogenic Ag-NPs were used in nematicidal assays. *Meloidogyne incognita* showed high mortality rates of 95.3%, 74.6%, and 72.6% after 72 h of treatment with the *X. indica* filtrate at three concentrations. At the same concentrations, biogenic Ag-NPs resulted in 82.0%, 90.0%, and 85.3% mortality rates, respectively. After 72 h, hatchability decreased by 53%, 74.6%, and 72.6% for the *X. indica* filtrate and 82.0%, 90.0%, and 85.3% for Ag-NPs. Quantitative real-time PCR (Q-PCR) revealed that Mi-Ache1 expression was lower in *M. incognita* second-stage juveniles (J2s) treated with the filtrate and Ag-NPs after 72 h compared to controls. Mi-Ache2 expression was also decreased, but only slightly. Furthermore, both the *X. indica* filtrate and biogenic Ag-NPs were safe in human lung (WI-38) and skin (HFB4) cell lines. These findings suggest that bacterial filtrates and their biogenic Ag-NPs could serve as cost-effective, environmentally friendly alternatives to commercial nematicides.

## 1. Introduction

Globally, plant parasitic nematodes, including root-knot nematodes (RKNs), result in crop losses of over $100 billion annually [1]. The most harmful nematode in the world is thought to be *Meloidogyne* spp. [2]. Root-knot nematodes, especially *Meloidogyne incognita*, pose a global threat to vegetable and field crops [3,4]. Second-stage juveniles (J2s), which can travel through soil and infect plants, are a critical in the life cycle of RKNs [5]. Since most nematicides have detrimental effects on the environment and public health, they are banned [6,7]. In many nematode–plant interactions, pre-plant soil nematode populations are the primary cause of yield loss [8]. *Meloidogyne incognita*, a harmful RKN species, significantly reduces crop yields globally [9,10,11]. Until now, the most commonly used methods for managing RKNs were chemical nematicides and resistant cultivars [12,13]. Chemical nematicides have generally been used to effectively manage RKNs [14], despite their demonstrated unfavorable side effects on humans, non-target organisms, and the environment [15]. However, as concerns about environmental safety and public health grew, several commonly used chemical nematicides were either discontinued or their usage was restricted. Thus, there is a need for more ecologically friendly ways to achieve control [16]. Because of their widespread distribution and frequent occurrence, RKNs must be effectively managed in order to keep their damage below thresholds.

The roots of crop plants can attract entomopathogenic nematodes (EPNs), which are also soil-dwelling nematodes, especially when host insect larva feed on the roots [17,18]. For the creation of biological control products to manage root arthropod pests, the two EPN families, *Steinernematidae* and *Heterorhabditidae*, have been extensively studied [19,20,21]. The genera *Steinernema* (Panagrolaimomorpha: *Steinernematidae*) and *Heterorhabditis* (Rhabditomorpha: *Heterorhabditidae*) contain entomopathogenic nematodes (EPNs) that have shown effective antagonistic effects against various insect pests [22,23,24]. These beneficial nematodes invade and eliminate insects within one to two days by releasing poisons from the symbiotic bacteria *Xenorhabdus* and *Photorhabdus*, which are carried into the hemocoel of their hosts, *Steinernema* and *Heterorhabditis*, respectively. The bacteria produce bioactive compounds (such as hemolysin, cytolysin, and toxins) that induce necrosis or apoptosis in host cells, which results in the death of the cells. A viable substitute for chemical control in the management of PPNs is the use of biological control agents (BCAs) [25,26]. The antagonistic effects of EPNs on PPNs have been observed and documented previously [18]. It has been demonstrated that EPNs can effectively control a variety of nematode species in both field and greenhouse settings, including *Rotylenchulus reniformis*, *Globodera rostochiensis* [27], *Belonolaimus longicaudatus* [28], *Meloidogyne* spp. [29,30], and *Criconemoides* spp. PPNs have been shown to have the greatest degree of control over RKNs. Applying EPN infective juveniles (IJs) from various strains has significantly reduced *Meloidogyne* spp. in terms of egg masses [31], the number of eggs [32], and second-stage juvenile (J2s) infectivity within the root matrix [28]. Additionally, symbiotic bacteria and/or their metabolites alone dramatically decreased host infectivity in greenhouse conditions [33] and significantly reduced RKN J2s in vitro [34]. *Xenorhabdus* and *Photorhabdus* are remarkable bacterial genera known for their ability to produce a wide range of secondary metabolites. These compounds include antimicrobial and insecticidal substances, making them promising candidates for bio-pesticide development. Additionally, they exhibit phase variation, allowing them to transition between different forms in response to environmental conditions. This adaptability enhances their effectiveness in biological control strategies and their potential applications in sustainable pest management.

These are Gram-negative bacteria that form symbiotic relationships with entomopathogenic nematodes, specifically *Steinernema* and *Heterorhabditis*. When these nematodes infect an insect host, they release the bacteria into the hemocoel, where they produce a range of toxins that contribute to the insect’s demise; a study conducted by [35] showed that the degree of control was similar to that of some chemical treatments. Additionally, with the use of bacterial supernatants from *Photorhabdus luminescens* and *Xenorhabdus* spp. [36], a significant nematicidal effect was reported for three Argentinean EPN isolates against *M. hapla*. The creation of bio-nanosilver, which is produced by entomopathogenic nematode symbiotic bacteria, is one innovative breakthrough that shows promise. Currently, bio-based nanosilver offers a sustainable and environmentally friendly alternative. Studies have shown that bio-based nanosilver can effectively target and eliminate *M. incognita*, while causing minimal harm to the ecosystem and non-target organisms. However, its performance against *Meloidogyne* species has not yet been extensively evaluated. Therefore, the main objective of this study is to investigate the nematicidal potential of biogenic bio-based nanosilver, derived from the symbiotic bacterium *X. indica*, to combat *M. incognita* under lab conditions. Furthermore, we aim to examine the expression of two neuronal acetylcholinesterase genes to determine their mechanism of action and lethality. In terms of biological safety, various cell lines were used to evaluate the cytotoxic effects of an *X. indica* cell-free filtrate and its biogenic Ag-NPs.

## 2. Materials and Methods

### 2.1. Collection of Samples

#### 2.1.1. Preparation of Root-Knot Nematode Inocula

To establish a pure population of *Meloidogyne incognita*, a single egg mass was isolated and allowed to hatch. The specimens were then reared on tomato plants (*Solanum lycopersicum* L.) in the greenhouse at the City of Scientific Research and Technological Application in Alexandria, Egypt. The experiments adhered to the guidelines set by the Egyptian Ministry of Agriculture, and commercial tomato seeds (cv. Alisa Co., El-Ekhlas, Kafer El- Sheikh, Egypt) were used. Eggs of *M. incognita* were retrieved from infested tomato roots using the sodium hypochlorite method described by [37]. The resulting egg suspension was kept at 25 ± 2 °C for three days to promote the hatching of second-stage juveniles (J2s). The experiments utilized freshly hatched J2s that were no more than 48 h old.

#### 2.1.2. Preparation of Culture Medium of Nematode Symbiotic Bacteria

The bacterium *Xenorhabdus indica* was isolated from the nematode *Steinernema abbasi*, with which it coexists [38]. To infect the larvae of *G. mellonella*, the concentration of infective juveniles (IJs) of *S. abbasi* was adjusted to 300 IJs/mL after being rinsed with sterile PBS buffer. After 48 h, the dead and infected *Galleria mellonella* larvae were collected so that the foregut could be detached with disinfected scissors. The hemolymph was retrieved using the double-tube centrifugation method.

Many minor pores were aligned at the bottom of a 1.5 mL sterilized microcentrifuge tube, and the *Galleria mellonella* larvae with their foreguts removed were placed into a 1.5 mL sterilized microcentrifuge tube. The hemolymph was extracted from the bottom of the 1.5 mL microcentrifuge tube after 60 s of centrifugation at 10,000 rpm. After being smeared on NBTA medium, the collected hemolymph was stored at 25 °C in a dark, constant-temperature incubator. Red colonies were selected and grown in fresh NBTA medium after 36–48 h to obtain the required nematode symbiotic bacteria.

The bacterial strains were cultivated on Luria–Bertani (LB) agar plates (15 g/L agar) and preserved in glycerol suspensions (50% *v*/*v*) at −80 °C. The *X. indica* strain was grown in LB broth (10 g/L tryptone, 5 g/L yeast extract, and 5 g/L NaCl pH 7.0) with shaking overnight at 30 or 37 °C. Subsequently, cultures were inoculated (1:100 *v*/*v*) in fresh LB medium and incubated for two days using a rotary shaker when necessary; additionally, 0.2% L-arabinose and the appropriate concentration of antibiotic (kanamycin 50 μg/mL) were added to the liquid culture.

#### 2.1.3. Identification of Symbiotic Bacterial Isolate

##### 16S rRNA Amplification and Sequencing

The 16S rRNA gene was amplified using universal bacterial primers: the forward primer was 5′-AGAGTTTGATCMTGGCTCAG-3′ and the reverse primer was 3′-TACGGYACCTTGTTACGACTT-5′ [39]. PCR mixtures were prepared in 0.2 mL sterile tubes with a total volume of 25 µL, containing 7.5 µL dH2O, 12.5 µL Master Mix (Itron Biotechnolog—Seoul, Republic of Korea), 1 µL DNA template, and 2 µL of each primer (10 pmol/µL). Amplification was performed in a thermal cycler (Eppendorf, Hamburg, Germany) with 30 cycles: initial denaturation at 95 °C for 2 min; 34 cycles of 95 °C for 40 s, annealing at 56 °C for 1 min, and extension at 72 °C for 1 min; and a final extension at 72 °C for 10 min. The products were stored at 4 °C [40]. PCR products were run on 1% agarose gel at 100 V for 45 min, stained with ethidium bromide, and visualized under the Gel Documentation System. The PCR products were purified using the PCR Clean Up Column Kit (Maxim Biotech Inc., Rockville, MD, USA) and sequenced with an automated DNA sequencer, the HiSeq-2000 (Macrogene Scientific Services Company, Seoul, Republic of Korea).

##### Phylogenetic Analysis Using the 16S rRNA Gene’s Obtained DNA Sequence

A phylogenetic analysis of sequence similarity was performed after multiple alignments with sequences of closely and highly related species using a BLAST search (https://blast.ncbi.nlm.nih.gov/Blast.cgi) Accessed on 1 June 2025. ClustalW (https://www.genome.jp/tools/clustalw/ accessed on 28 December 2025) in BioEdit v7.2.5 software was used to perform the comparative analysis. The phylogenetic tree was built using the neighbor-joining (NJ) method, and its similarity to other bacterial species was evaluated using BLAST and Evolutionary Genetic Analysis version 7 (MEGA 7) software [41]. For the DNA BLAST search, the other bacteria that were used to construct the phylogenetic tree were chosen because they were highly similar to our isolate (more than 96%).

### 2.2. Synthesis of Biogenic Silver Nanoparticles (Ag-Nps)

Silver nitrate (AgNO_3_, 99.9%, with an average molecular weight of 169.87) and acetone, used as a solvent, were sourced from Lab Chemicals Trading Company, Egypt. As described above, to evenly coat AgNO_3_, 90 mL of *X. indica* bacterial filtrate was combined with 10 mL of AgNo3 (1 mM) solution using a magnetic stirrer and heater. The mixture was then incubated in a rotary shaker at 150 rpm, kept in the dark at 29 °C, and allowed to react for 24 h; the filtrate was centrifuged at 9000× *g* for 30 min, and a control sample lacking silver ions was also prepared. The precipitate was collected, washed three times with sterile distilled water, dried in an oven at 50 °C for 24 h, weighed, and stored in the dark until use. For the positive control, 1 mM AgNO_3_ (10 mL) was dissolved in 20 mL of deionized water. The bacterial strain’s ability to synthesize Ag-NPs was tested by observing visible color changes and analyzing samples with UV–visible spectroscopy with three replicates; the experiment was repeated twice. In addition, characterization of antimicrobial activity was performed using Fourier transform infrared spectroscopy, transmission electron microscopy, and scanning electron microscopy.

#### Uv-Vis Spectrophotometer

The bacterial filtrate and AgNO_3_ mixtures were analyzed at room temperature for UV–visible absorption using a T60 Visible Spectrophotometer (PG Instruments Limited- Leicestershire, UK) at 450 nm; the absorbance of silver nanoparticles was measured.

### 2.3. Characterization of Biogenic Silver Nanoparticles

#### 2.3.1. Transmission Electron Microscopy (TEM)

TEM (JEM-100CX; JEOL Ltd., Tokyo, Japan) running at an accelerating voltage of 80 kV was used to observe the particle’s size and shape. Deionized water was used to dilute the prepared silver suspensions tenfold. A coated copper grid was sprayed with a drop of the suspension, which was then left to dry at room temperature.

#### 2.3.2. Microscopy (SEM)

The color and physical structure of the synthesized biogenic Ag-NPs were confirmed using a scanning electron microscope (SEM). The chemically synthesized Ag-NPs were held at 8000× *g* for 20 min at 4 °C, cleaned with absolute ethanol, and fixed with 2% glutaraldehyde and 1% osmium tetroxide (OsO4). After being fixed, the Ag-NP samples were cleansed, transported to ethanol, and subsequently dried in different concentrations of the ethanol (50, 75, and 100%). The dried Ag-NPs were coated with a fine coating of gold. Using SMILE VIEW software and a JEOL Ltd., Tokyo, Japan), the particle size of Ag-NPs was determined by randomly selecting particles from samples.

#### 2.3.3. Fourier Transforms Infrared Spectroscopy (FT-IR) Analysis

The biomolecules in the bacterial filtrate responsible for biogenic Ag-NP formation were analyzed using an FT-IR spectrometer (FT-IR-8400S, Shimadzu, Japan). To identify the composition of the dried Ag-NPs, they were pressed into thin pellets with potassium bromide (KBr) powder and scanned across wavelengths from 400 to 4000 cm^−1^.

### 2.4. Laboratory Assessment of Bacterial Filtrate (Cell-Free) and Its Biogenic Silver Nanoparticle Activity

#### 2.4.1. Mortality of Second-Stage Juveniles (J2s) of *M. incognita*

The nematicidal effect of symbiotic *X. indica* biosynthesized Ag-NPs on *M. incognita* J2 mortality was assessed in a laboratory experiment. The biosynthesized Ag NPs were administered to the second-stage juveniles at varying concentrations (S = 9 mL biogenic Ag-NPs + 1 mL J2s suspension, S/2 = 4.5 mL biogenic Ag-NPs + 4.5 mL dH2O + 1 mL J2s suspension, and S/3 = 2.25 mL Ag-NPs + 6.75 mL distilled water + 1 mL J2 suspension). Each treatment was represented by approximately 100 newly hatched J2s per milliliter during the bioassay, which was conducted in cell culture plates. The plates served as controls and contained 1 mL of nematode suspension and 9 mL of dH_2_O. Two sets of assays each with five replications were employed, and the experiment was repeated twice, utilizing the commercial bionematicide Anti-Nema^®^ as a reference nematicide. J2 mortality was measured at 12, 24, and 72 h after exposure; the plates were incubated at 25 ± 2 °C, and the nematodes were deemed dead if they showed no signs of movement in distilled water. The percentage of mortality was determined using the formula of Abbott [42].

Mortality % = (No. of alive J2s in control − No. of alive J2s in treatment)/(No. of total alive J2s in control) × 100

#### 2.4.2. Hatchability of *M. incognita* Eggs

The bioassay to evaluate the nematodes’ egg hatchability was conducted using a sterile 6-well cell culture *plate* (SPL Life Sciences Co., Ltd., Pocheon-si, Republic of Korea), with approximately 100 eggs in each well. Three different concentrations (S1, S2, and S3) of bacterial filtrate were tested, along with Anti-Nema^®^ commercial bionematicide, and dH_2_O served as the control. Five replicates were prepared for each treatment, and each experiment was repeated at least twice. All treatment plates were incubated at 27 ± 2 °C. Egg hatching was recorded at 24 and 48 h. After each measurement, eggs were washed with 1 mL of dH_2_O and transferred to new plates containing fresh solutions of the same concentration. The egg hatch inhibition percentage was calculated according to [27].

Hatchability % = (No. of egg in control − No. of egg in treatment)/(No. of total egg in control) × 100

### 2.5. Detection of Gene Expression of Root-Knot Nematode

The J2s larvae of *M. incognita* were combined with 750 μL of Isol-RNA Lysis reagent at room temperature and vortexed vigorously for 15 s. This mixture was then left at room temperature for 5 min. To eliminate insoluble material, the homogenate underwent centrifugation at 9000× *g* for 15 min at 4 °C. The resulting clear homogenate was transferred to a clean tube, and 200 μL of chloroform was added. The mixture was vortexed for 15 s at room temperature, incubated for 5 min, and then centrifuged at 8000× *g* for 15 min at 4 °C. The upper aqueous phase was carefully transferred to a new 2 mL centrifuge tube, combined with an equal volume of isopropanol, and inverted five times. After 10 min of incubation at room temperature, double the volume of 70% ice-cold ethanol was added, and the sample was stored at −20 °C for 90 min. Following this, the tube was centrifuged at 9000× *g* for 12 min at 4 °C, and the supernatant was gently removed. The RNA pellet was washed with 1 mL of 70% cold ethanol and centrifuged again at 9× *g* for 6 min at 4 °C before being dried. RNA was reconstituted in 50 μL of RNase-H_2_O, and its concentration and purity were evaluated by measuring absorbance at 260 nm (A260) and 280 nm (A280). Samples with an A260/A280 ratio of 1.8 or greater were selected for further analysis.

For cDNA synthesis, 2 μg of total RNA and 1 μL of oligo dT primer were combined with nuclease-free H_2_O to a final volume of 12 μL, incubated at 65 °C for 5 min, and then cooled on ice. The reaction was then augmented with 4 μL of 5× reaction buffer, 1 μL of RNase inhibitor, 2 μL of dNTP mix, and 1 μL of reverse transcriptase (or nuclease-free H_2_O for a negative control). This mixture was briefly spun down and incubated at 42 °C for 60 min, followed by heat inactivation at 70 °C for 5 min.

To conduct qPCR analysis, 12.5 μL of 2× SYBR Green Master Mix was mixed with 5 μL of cDNA and specific primer sets (Table 1) for MI-Ach1 and MI-Ach2. The housekeeping gene was represented by 0.5 μL of 10 pmol/mL forward and reverse primers. The total reaction volume was brought to 25 μL with nuclease-free water, briefly spun down, and loaded into the thermal cycler. The qPCR program included initial denaturation at 95 °C for 10 min, followed by 40 cycles of denaturation at 95 °C for 15 s, annealing at 60 °C for 30 s, and extension at 72 °C for 30 s. The Ct values obtained were utilized to assess gene expression levels, with the fold change calculated using the following formula: Fold change = log (2 − ΔΔCT).

### 2.6. Cytotoxicity Determination

A human lung fibroblast cell line (WI-38, passage number 31) and a human skin fibroblast cell line (HBF4) were utilized to investigate the toxicity of the *X. indica* filtrate and its biogenic Ag-NPs in comparison to Anti-Nema. The American Type Culture Collection (ATCC) supplied the cell lines used in this study. Cells were cultivated in DMEM medium containing 10% fetal bovine serum, seeded in sterile 96-well culture plates (1 × 104 cells/well), and then incubated for 24 h at 37 °C in a CO_2_ incubator. After 24 h of cell attachment, Wi-38 or HBF4 cells were incubated with twofold serial dilutions of the tested compounds for 72 h. Cell viability was assessed using MTT in accordance with the Mosmann method [45,46]. MTT (0.5 mg/mL) was added to each well, and the plate was incubated at 37 °C for 3 h in a CO_2_ incubator. Following the removal of the MTT solution and the addition of 100 μL of DMSO, the absorbance of each well at 570 nm was measured via a microplate reader (BMG LabTech, Ortenberg, Germany) for calculation of the percentage of cell viability (absorbance of the treated wells/absorbance of untreated control × 100). The concentrations of the three tested materials (IC50 and EC100) at 50% and 100% cell survival were estimated using GraphPad Instat [47].

### 2.7. Data Analysis

SAS version 9.4 (SAS Institute Inc., Cary, NC, USA) was used to conduct a two-way analysis of variance (treatments and times) on in vitro data. Duncan’s multiple range test separated treatment means with a 5% chance. Significant differences in gene expression and enzyme activity were identified using Duncan’s multiple range tests (*p* ≤ 0.05) and SPSS software (Version 16.0) [48].

### 2.8. Molecular Data Analysis

To determine gene expressions using β-actin (housekeeping gene) and two other Mi-ACh genes, the (ΔΔCq) expression values were calculated for RNA samples of J2s treatment: ΔCq = Cq − reference gene; ΔΔCq = Cq − Control; ΔΔCq expression = 2 (−ΔΔCq). The relative expression ratio for the real-time PCR is represented mathematically by the equations. In contrast to the reference gene, the target gene’s ratio is expressed in terms of J2s. The data were statistically evaluated, interpreted, and analyzed using Rotor-Gene-6000 version 1.7.

## 3. Results

### 3.1. Molecular Identification of Symbiotic Bacteria Strains

**Figure 1:**
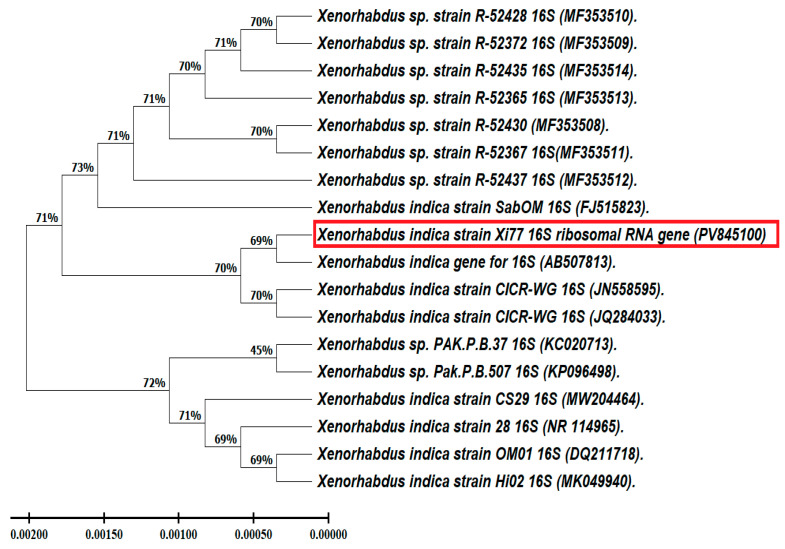
*Xenorhabdus indica’s* phylogenetic analysis based on gene sequences illustrating its relationships to other *Xenorhabdus* species.

Based on morphological features like color and spore shape, the bacterial isolate was initially identified. Amplification and sequencing of bacterial rRNA genes resulted in a 1530 bp nucleotide sequence, which has been submitted to NCBI GenBank under Accession Number PV845100 for the *X. indica* strain Xi 77. This process confirmed its identification as *Xenorhabdus indica*. The phylogenetic analysis revealed that the *X. indica* isolate Xi 77 has a close relationship with *Xenorhabdus indica* (AB507813), as illustrated in Figure 1. The phylogenetic tree was constructed using the maximum parsimony method with MEGA version 7.

### 3.2. Characterization of Biogenic Ag-Nps

#### 3.2.1. Uv–Vis Spectral

**Figure 2:**
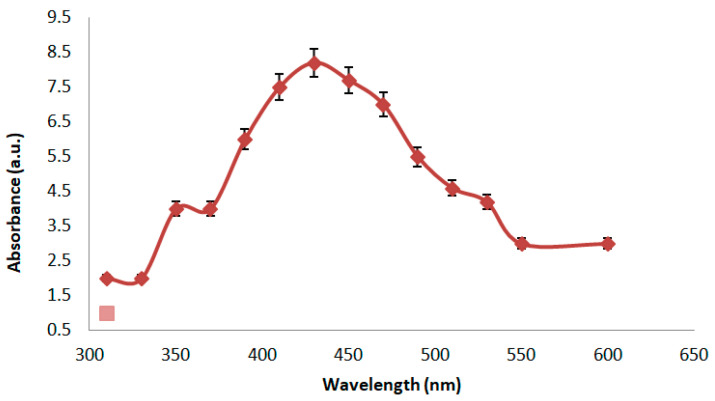
UV spectra of biogenic Ag-NP.

It is well established that a brown coloration indicates the presence of biogenic Ag-NPs. Aqueous solutions containing Ag-NPs appear clear, yellowish, or light brown due to surface plasmon resonance (SPR) within the particles. In our study, the Ag-NPs were produced by exposing *X. indica* extract to an AgNO_3_ solution. The complete reduction of Ag ions was achieved during 2 h of incubation. Visual observation verified the formation of silver nanoparticles. The presence of surface plasmon resonance (SPR) is indicated by a distinct peak in the UV–vis absorption spectrum of Ag-NPs synthesized using *X. indica*, which appears at 480 nm. After two hours of reaction, a clear peak at 430 nm was seen in the UV–vis absorption spectrum, and its intensity increased steadily with reaction time. This absorbance peak was visible in UV–visible absorption spectra, suggesting surface plasmon resonance (SPR). Regarding the bacterial filtrate and its powder, the formation of Ag-NPs was very rapid, within 2 min and 2 h, respectively, and they maintained their color stability for an extended period at room temperature (Figure 2).

#### 3.2.2. Transmission and Scanning Electron Microscopy (TEM and SEM)

**Figure 3:**
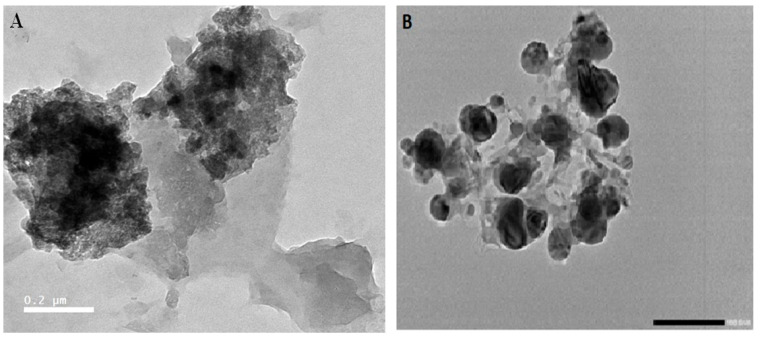
(**A**) SEM micrograph of AGNPs’ morphology at 10 µm resolution. (**B**) TEM micrograph.

The distinguishing size details of the biogenically synthesized nanoparticles from symbiotic *X. indica* were characterized and represented by transmission and scanning electron microscopy (TEM and SEM), as shown in Figure 3A,B. It is clear from the micrograph that separate silver nanoparticles, as well as aggregates, are present, and those prepared from the *X. indica* filtrate are spherical or oval-shaped.

#### 3.2.3. FT-IR Analysis

**Figure 4:**
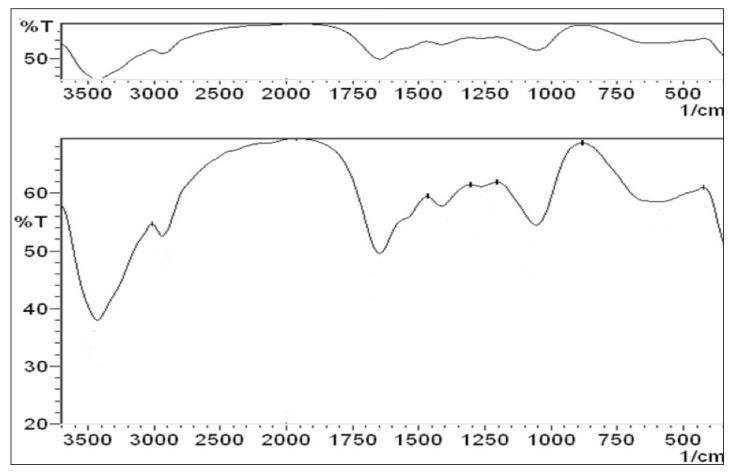
FT-IR spectra of biogenic Ag-NPs with *X. indica* bacteria ranging 3500–500 cm^−1^; T: transmittance.

The FTIR analysis results indicate various bond stretches at different peaks. In Figure 4, the peak at (1263.42 cm^−1^) corresponds to the C–N stretch in aromatic amines, while the peak at (590.24 cm^−1^) relates to the C–Br stretch in alkyl halides. Peaks at (1055.10 cm^−1^) and (1076.32 cm^−1^) are associated with the C–N stretch in aliphatic amines.

### 3.3. Toxicity of Symbiotic X. indica’s Bacteria and Their Biogenic Ag-Nps on Second-Stage Juveniles (J2s) of M. incognita

**Figure 5:**
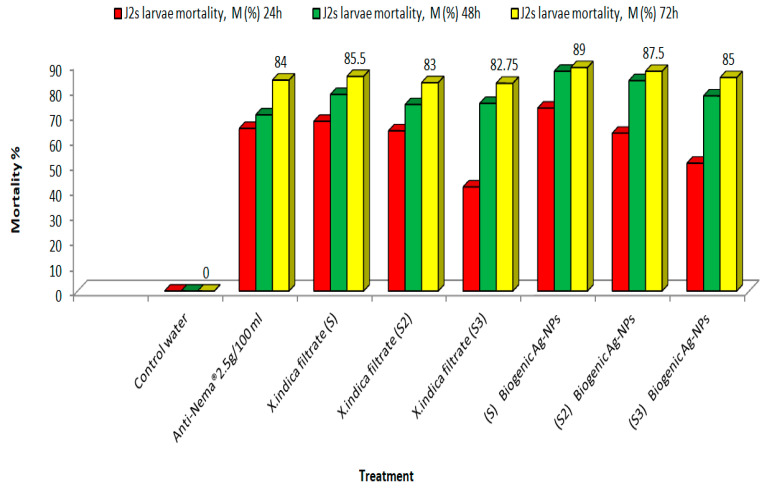
The effects of the *X. indica* filtrate and its biogenic Ag-NPs compared to a commercial anti-Nema bio-pesticide on J2 mortality percentage (M%) among *Meloidogyne incognita* following exposure for 24, 48, and 72 h.

**Figure 6:**
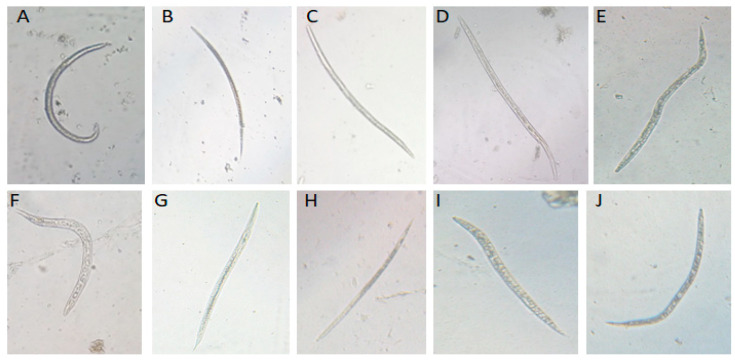
*M. incognita* mortality of J2s under a light microscope following 72 h immersion in the *X. indica* filtrate compared with two controls, (**A**) H_2_O and (**B**) Anti-Nema, (**C**) at 100%, (**D**) 75%, and (**E**) 50%; furthermore, its biogenic Ag-NPs were compared with two controls, (**F**) H_2_O and (**G**) Anti-Nema, (**H**) at 100%, (**I**) 75%, and (**J**) 50%.

The toxicity of nematode symbiotic bacteria and their biogenic Ag-NPs at three concentrations against *M. incognita* J2s was evaluated through immersion with incubation intervals, as depicted in Figure 5 and Figure 6, indicating concentration-dependent toxicity. The data show a definite positive relationship between the bacterial concentration and the observed mortality percentage of *M. incognita* J2s: mortality percentages increased with increasing biogenic NP concentration. At the highest concentration (S%), the mortality percentage of *X. indica* treatment reached 85.5% after 72 h, while exposure to the biogenic Ag-NPs resulted in 89% mortality after the same duration. This percentage significantly increased over time, suggesting a prolonged and cumulative toxic effect. Furthermore, when exposed to sterile water, the mortality of *M. incognita* J2s was negligible. However, the mortality rates of *M. incognita* J2s treated with the *X. indica* filtrate increased as the concentration rose after 72 hat the S concentration. In contrast, the mortality rate of *M. incognita* J2s treated with biogenic Ag-NPs was significantly higher compared to treatment with the *X. indica* filtrate at all tested concentrations and exposure times.

### 3.4. Toxicity of Symbiotic X. indica’s Bacteria and Their Biogenic Ag-Nps on Hatchability of M. incognita Egg

**Figure 7:**
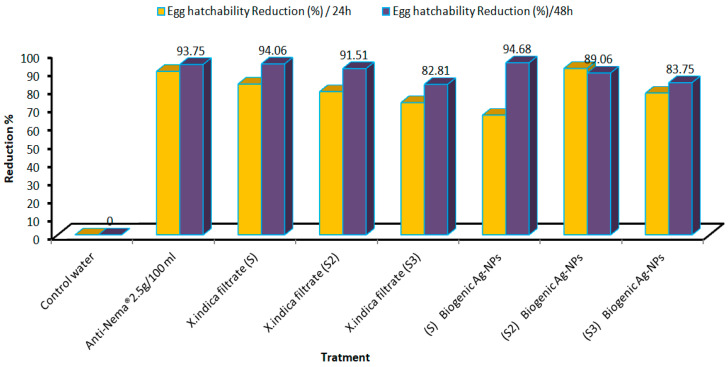
The effects of the *X. indica* filtrate and its Ag-NPs compared to a commercial anti-Nema bio-pesticide on the reduction percentage (R%) of *M. incognita* egg hatchability following exposure for 24 and 48 h.

The toxicity of nematode symbiotic bacteria and their biogenic Ag-NPs at three concentrations against *M. incognita* eggs was evaluated through immersion with incubation intervals, as shown in Figure 7. The data indicate concentration-dependent toxicity, revealing a clear negative relationship between bacterial concentration and the hatchability of *M. incognita* eggs, with hatching rates decreasing as biogenic Ag-NP concentration increased. At the highest concentration (20%), egg hatchability decreased to 94.68% after 72 h of exposure. This percentage significantly decreased over time, suggesting a prolonged and cumulative toxic effect. In the case of the *X. indica* filtrate, an egg hatchability rate of 94.06% was recorded after 48 h.

### 3.5. Quantitative Real-Time PCR

To gain insight into the mechanism of the innate response system of *M. incognita* upon immersion exposure to the *X. indica* bacterial filtrate and its biogenic Ag-NPs, the relative transcript expression levels of acetylcholinesterase (Mi-AChE-1 and Mi-AChE-2) genes were detected in J2 larvae using quantitative real-time PCR.

**Figure 8:**
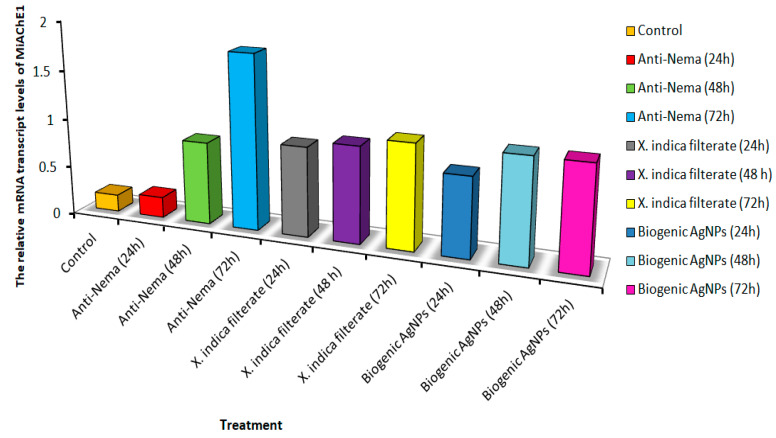
QRT-PCR confirmation of the relative gene expression of MiAchE1 in *M. incognita* J2 larvae after treatment with the *X. indica* filtrate, compared to healthy controls and those treated with a commercial anti-nematode bio-nematicide for 24, 48, and 72 h.

**Figure 9:**
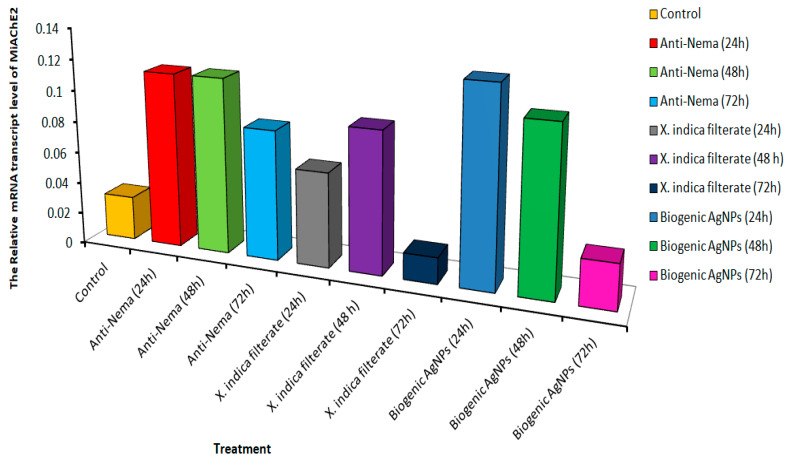
QRT-PCR confirmation of the relative gene expression of MiAchE2 in *M. incognita* J2 larvae after treatment with the *X. indica* filtrate, compared to healthy controls and those treated with a commercial anti-nematode bio-nematicide for 24, 48, and 72 h.

The impact of the symbiotic bacterium *X. indica* filtrate and its biogenic Ag-NPs on the targeted genes of *M. incognita* J2s was measured after exposure for 12, 24, and 72 h at the S% concentration, during which time the nematodes were eliminated. Since variations in gene expression can be used to diagnose the effects of environmental chemical exposure, we used q-PCR to assess the expression of two genes (Mi- AChE-1 and Mi-AChE-2) involved in motility and chemoreception mechanisms. After 72 h of incubation, Mi-AChE-1 expression increased in nematodes treated with commercial nematicides, followed by increases with *X. indica* and its biogenic Ag-NPs after 48 and 72 h, respectively. However, after 12 h of exposure to both *X. indica* and its biogenic Ag-NPs, the expression levels changed only slightly (Figure 8). While the higher expression levels with biogenic Ag-NPs after 24–48 h decreased after 72 h, the relative expression of Mi-AChE-2 in J2s incubated with *X. indica* increased at 42 and 48 h and returned to basal levels after 72 h. In J2s treated with Anti-Nema, the level remained higher than in the untreated control but was about three times lower than that observed in the biogenic Ag-NP treatment (Figure 9). We conclude that Mi-AChE-1 and Mi-AChE-2 respond to treatments with *X. indica* and biogenic Ag-NPs. They also show a higher expression pattern than Anti-Nema, indicating that they affect the nervous system similarly, leading to juvenile nematode mortality.

### 3.6. Effect on Human Normal Cell Proliferation

**Figure 10:**
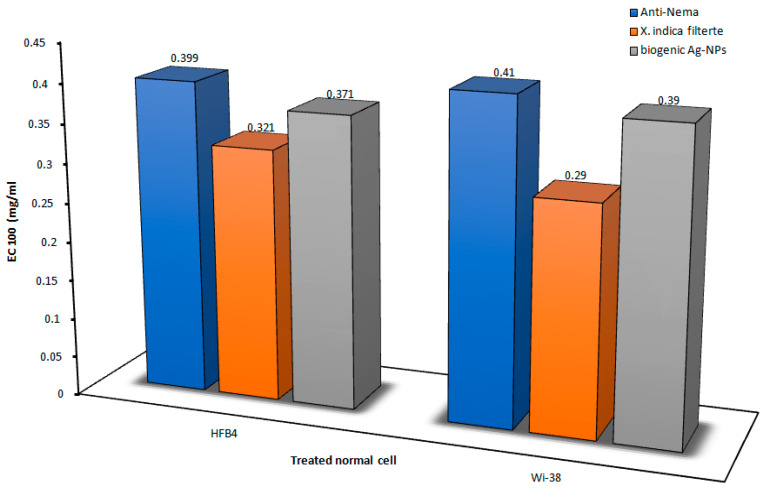
The cytotoxicity of the *X. indica* filtrate and its Ag-NPs was demonstrated at a dose causing 50% cell death (IC50) on both human normal cell lines (HFB4 and WI-38), compared to Anti-Nema.

**Figure 11:**
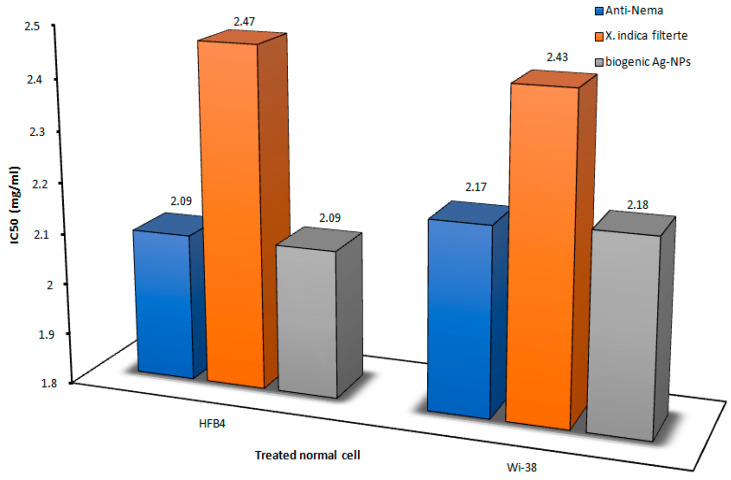
The cytotoxicity of the *X. indica* filtrate and its Ag-NPs was observed at a safe dose (EC100) on both human normal cell lines (HFB4 and WI-38), in comparison to Anti-Nema.

The *X. indica* filtrate and its biogenic Ag-NPs had higher IC50 and EC100 values (≥2 and ≥0.15 mg/mL) than the Anti-Nema commercial bionematicide, as illustrated in Figure 10 and Figure 11. This suggests that the tested compounds were less harmful to the growth of normal human cell lines (WI-38 and HFB4) than this common nematicide.

## 4. Discussion

Our findings that both the *X. indica* filtrate and biosynthesized Ag-NPs significantly increased *M. incognita* J2 mortality and inhibited egg hatching are in agreement with previous studies demonstrating strong nematicidal effects of bacterial culture filtrates on root-knot nematodes. Treatment with cell-free culture filtrates from entomopathogenic bacteria including *Xenorhabdus* showed pronounced toxicity against *M. incognita* juveniles in vitro, with mortality rates increasing over time and with higher filtrate concentrations, indicating a clear dose- and time-dependent relationship [49]. Similarly, our results showed that the *X. indica* filtrate and Ag-NPs caused J2 mortality and inhibited egg hatching of *M. incognita* after 72 h of exposure. This aligns with [50], who reported that *C. elegans* was killed by *Xenorhabdus* bacterial cell-free culture supernatants, potentially due to protein toxins or nanoparticles (NPs). Additionally, they found that the nematicidal compounds produced by these three *Xenorhabdus* spp. are heat-stable, consistent with small-molecule NPs, rather than toxic proteins. Although toxic proteins could have a similar effect, ref. [51] demonstrated that *Xenorhabdus* can rapidly kill *C. elegans* by producing NPs in their culture supernatants. Essentially, cell death is primarily driven by decreased membrane permeability and disruption of the proton motive force, resulting in cellular dysfunction [52]. Similarly, silver nanoparticles produced through green biosynthesis methods have been reported to exhibit potent nematicidal activity against *M. incognita*. In a recent in vitro study, biosynthesized Ag-NPs caused substantial immobilization and mortality of juvenile nematodes at low concentrations, with effects comparable to or exceeding those observed with conventional treatments [53]. In previous studies, scientists emphasized that these toxin complexes, once released into the host’s hemolymph, induce histopathological damage and septicemia, ultimately causing fatality [54,55]. The majority of *Xenorhabdus* bacteria demonstrated potent nematode activity against the *C. elegans* L4 stage in the microtiter *plate* nematicidal assay [51]. Our findings align with earlier results that have demonstrated nematicidal activity in cell-free culture supernatants of *Xenorhabdus* and *Photorhabdus* against J2s of *M. incognita*. Nematode mortality might occur due to nanoparticles binding to intracellular sulfur-containing proteins or phosphorus in DNA, leading to degradation of organelles and enzymes [56].

By inhibiting phospholipase A2, the entomopathogenic bacteria have a nematicidal effect during infection, impairing the nematode and insect resistance. Additionally, the bacteria suppress the expression of genes responsible for antibacterial peptides, thereby weakening the insect pest’s humoral immune response [57].

Furthermore, *Photorhabdus* and *Xenorhabdus* produce a range of insecticidal toxins, including toxin complexes (Tc’s), the Making Caterpillar Floppy (Mcf) toxin, binary toxins PirAB, juvenile hormone esterase (JHE), and ureases, which exert entomotoxic effects independently of ureolysis [58,59,60]. Ref. [61] discovered that ammonium produced by *Xenorhabdus* bacteria has nematicidal effects on *M. incognita* J2s.

Class C AChE has been linked to the resistance mechanisms of OPs and CB26 in RKN *M. incognita* and *M. arenaria*. According to [62], soluble AChE is essential to the nematodes’ chemical defense mechanisms against a variety of xenobiotics in *B. xylophilus*. The high threshold of insensitivity to populations of *M. incognita* that are resistant to fosthiazate may therefore be due to the enzyme’s differential biochemical sensitivity or to a modified form of AChE that is resistant to inhibitors [45,63].

ACE1 is involved in synaptic transmission in *C. elegans*, even though the functional distinction between ACE1 and ACE2 in nematode AChEs remains unclear [64]. According to earlier findings, ACE2 may be involved in feeding, reproduction, and other behaviors in addition to playing important roles in synaptic transmission [62]. Nematodes, on the other hand, showed ACE2 expression patterns that varied by species and stage. ACE2 was primarily found in the head and tail ganglion regions of *C. elegans* and was primarily expressed in the infectious juveniles of *G. pallid* [65,66]. In *M. incognita*, ACE2 was expressed in J2 both before and after hatching in both males and females. It has been suggested that this gene contributes to the pharyngeal valve’s contraction during feeding [67]. However, the infection of *M. incognita* is not significantly affected by the RNAi-mediated gene silencing of ace2, indicating that either ace1 compensates for the RNAi effect of ace2 or that the RNAi of ace2 has no detrimental effect on nematode feeding and infection. The interaction of ace1 and ace2 in synaptic transmission and other motor behaviors was confirmed by quantitative RT-PCR analyses, which revealed that the RP population’s ace1 transcription level was lower than that of ace2 [45].

Analysis of inhibition activity showed that fosthiazate, an OP nematicide known for its strong anti-AChE properties effective against various mammals and insects, suppressed AChE in *M. incognita* [68]. Since both ACE1 and ACE2 are suggested as the main postsynaptic ACEs in plant parasitic nematodes, intense inhibition of these enzymes could result in high toxicity from nematicides. Studies on the inhibitory effects of OPs and CBs on the three AChEs of the pinewood nematode *Bursaphelenchus xylophilus* revealed different inhibition patterns: BxACE1 was less affected by OPs but more sensitive to CBs compared to BxACE2 [69]. The ACE active site gorge contains many aromatic residues, which affect the binding affinity of substrates or inhibitors.

In conclusion, we found that *X. indica* bacteria and their biogenic nano-silver caused high mortality in the destructive root-knot nematode *M. incognita* by affecting some of the studied genes, including the acetylcholinesterase genes Mi-AChE-1and Mi-AChE-2, which paralyzes nematodes and leads to their death when exposed to the bacterium filtrate or nano-silver after 72 h of exposure. We evaluated the toxicity of the *X. indica* cell-free filtrate and its biogenic Ag-NPs on two cell lines (WI-38 and HBF4) to ascertain whether employing such particles as a pesticide against plant parasitic root-knot nematodes is appropriate.

The EC100 values of Ag-NPs were 1.78 and 4.23 μg mL^−1^ for WI-38 and HBF4, respectively, when a cell line was used as a test subject to assess the cytotoxic potential of the *X. indica* cell-free filtrate and its biogenic Ag-NPs [49].

The cytotoxicity values (IC50 and EC50) of these nanoparticles were at least twice as high as those of the conventional insecticide, based on the current results (Figure 10 and Figure 11). The growth of both normal cell lines was found to be more safely impacted by the *X. indica* cell-free filtrate and its biogenic Ag-NPs. Similarly [70], when tested on normal human skin fibroblasts and blood lymphocytes, the biosynthesized Ag-NPs showed IC50 values of less than 0.3 and 8.21 mg/mL, respectively. With no detrimental cytotoxic effect and IC50 values that were noticeably higher than its MBC values, it is safe to use at its biocidal concentration. An eco-friendly *X. indica* cell-free filtrate and its biogenic Ag-NPs were described, featuring easy scalability and non-toxic by-products, making it a powerful and nematicidal option. The choice of lung- and skin-specific cell lines for the cytotoxicity assay was based on their direct relevance to the most likely exposure routes during pesticide handling and application. Lung epithelial cells serve as established in vitro models for evaluating respiratory toxicity due to inhalation exposure, capturing key cellular responses to airborne substances, while skin cell models are widely used to investigate dermal toxicity resulting from direct contact exposure. The use of these in vitro models aligns with existing toxicological research that applies human lung and skin cell lines to assess cytotoxic responses to environmental and chemical stressors, enabling human-relevant safety evaluations for exposure pathways of concern [71,72,73]. Additionally, best practice guidance such as ISO 10993-5 for in vitro cytotoxicity emphasizes the importance of selecting cell lines that are relevant to the target tissue or organ for reliable in vitro toxicity data [74].

## 5. Conclusions

In conclusion, *Xenorhabdus* produced active compounds, in addition to biosynthesized biogenic Ag-NPs, that exhibit strong nematicidal activity and exert a direct effect on acetylcholinesterase activity in the juveniles of *M. incognita*, leading to nematode mortality upon exposure to *X. indica* filtrates or Ag-NPs. Moreover, such microbially produced nanoparticles are, in principle, biodegradable and eco-friendly for agricultural applications, making them potentially useful for the biocontrol of nematodes in crop and vegetable production. Furthermore, the mechanism of action of these promising nematicidal compounds is linked to acetylcholinesterase genes, which were studied through the quantification of up- and down-regulation using Q-PCR. Therefore, our findings may open new avenues for the development of efficient and safe nematicidal compounds that can be used to enhance crop quality production.

## Figures and Tables

**Table 1:** Quantitative real-time PCR primers.

Primers	Primer Sequence 5′→3′	Reference
Mi-AChE1-F	ATGATGGATTATTCAATAGAGGACAG	[43]
Mi-AChE1-R	CTATTTTATTCCACAAACATCATTATCACC	[44]
Mi-AChE2-F	GTGGATCCGTTGACGTTCTTA	[45]
Mi-AChE2-R	ACGTCTAACCAAATGAGCAATAAC	[46]
q-Actin-F	GGGTATGGAATCTGCTGGTAT	[45]
q-Actin-R	AGAAAGGACAGTGTTGGCGTA	[45]

## Data Availability

The sequence of *Xenorhabdus indica* was deposited at NCBI GenBank (Accession Number: PV845100) for the *X. indica* strain Xi 77 (*Xenorhabdus indica* strain Xi77 16S ribosomal RNA gene, partial sequence—Nucleotide—NCBI).
